# Patients from general practice with non-specific cancer symptoms: a retrospective study of symptoms and imaging

**DOI:** 10.3399/BJGPO.2023.0058

**Published:** 2024-01-10

**Authors:** Jonas Michele Dorph de Chiffre, Tina Elisabeth Ormstrup, Martin Weber Kusk, Søren Hess

**Affiliations:** 1 Faculty of Health Sciences, University of Southern Denmark, Odense, Denmark; 2 Department of Radiology and Nuclear Medicine, University Hospital of Southern Denmark, Esbjerg, Denmark; 3 IRIS - Imaging Research Initiative Southwest, University Hospital of Southern Denmark, Esbjerg, Denmark; 4 Radiography & Diagnostic Imaging, School of Medicine, University College Dublin, Dublin, Ireland; 5 Department of Regional Health Research, Faculty of Health Sciences, University of Southern Denmark, Odense, Denmark; 6 Department of Nuclear Medicine, Odense University Hospital, Odense, Denmark

**Keywords:** non-specific cancer symptoms, imaging, general practice, Denmark, retrospective studies

## Abstract

**Background:**

Patients with non-specific symptoms or signs of cancer (NSSC) present a challenge as they are a heterogeneous population who are not candidates for fast-track work-up in an organ-specific cancer pre-planned pathway (CPP). Denmark has a cancer pre-planned pathway for this population (NSSC-CPP), but several issues remain unclarified, for example, distribution and significance of symptoms and findings, and choice of imaging.

**Aim:**

To investigate symptoms, cancer diagnoses, and diagnostic yield of computed tomography (CT) and fluorine-18 fluorodeoxyglucose positron emission tomography/computed tomography (^18^F-FDG-PET/CT) in patients on NSSC-CPP to improve the overall diagnostic process.

**Design & setting:**

A retrospective medical chart review in a 1-year consecutive cohort (2020).

**Method:**

A total of 802 referrals were reviewed for diagnostic imaging in patients with NSSP from general practices, specialist practices, or the local hospital diagnostic centre responsible for NSSC-CPP.

**Results:**

The study included 248 patients; 21% had cancer, most frequently gastrointestinal cancer (27%). The most frequent symptom was weight loss (56%). CT had a sensitivity of 85%, specificity of 87%, positive predictive value (PPV) of 65%, and negative predictive value (NPV) of 96%. For ^18^F-FDG-PET/CT, the numbers were sensitivity 82%, specificity 62%, PPV 33%, and NPV 94%. Patients frequently underwent subsequent examinations following initial imaging.

**Conclusion:**

The findings were in accordance with the literature. Patients with NSSC had a cancer prevalence of 21%, most frequently gastrointestinal. The most frequent symptom was weight loss and, even as the only symptom, it is a potential marker for cancer. CT and ^18^F-FDG-PET/CT were sensitive with high NPV, whereas PPV was superior in CT. Better stratification by symptoms or findings is an obvious focus point for future studies to further optimise the NSSC-CPP work-up strategy.

## How this fits in

Patients with non-specific signs or symptoms of cancer (NSSC) are a heterogeneous and clinically challenging group in general practice. A significant proportion is diagnosed with underlying cancer, but the most suitable diagnostic strategy for general practice remains controversial. This study found a 21% cancer prevalence, most frequently gastrointestinal, and symptoms and signs reflected the literature, with weight loss as the most prevalent one. The current strategy with CT of chest and abdomen or ^18^F-FDG-PET/CT performed well in this population with high sensitivity and negative predictive values (NPV) but more moderate specificity and positive predictive values (PPV), especially for ^18^F-FDG-PET/CT.

## Introduction

Correct and timely treatment of cancer requires a fast and accurate diagnostic strategy. Recognising relevant symptoms is the first step, but only half of patients with cancer present with alarm symptoms.^1,2^ Fifteen per cent of the general population experience alarm symptoms during a year,^3,4^ whereas annual cancer incidence is much lower; for example, <1% in Denmark.^5^ The diagnostic process is even more difficult if the cancer presents with NSSC for example, general malaise, fatigue, or weight loss.^2,6,7^ These challenges were revisited by a recent analysis paper.^8^

In the late 1990s, cancers were diagnosed later in Denmark than in other European countries, and the mortality rates were higher.^7,9–11^ As a result, the Danish Health Authority introduced national cancer pre-planned pathways (CPP) to ensure fast work-up^7,12^ for specific cancer suspicions and alarm symptoms, and also for NSSC (NSSC-CPP).^13,14^ Patients with NSSC are investigated in general practice or hospital-based diagnostic centres.^12^ Initial work-up usually includes clinical examination, standard blood tests, and diagnostic imaging; usually either CT of chest and abdomen or a positron emission tomography with ^18^F-labelled fluorodeoxyglucose (^18^F-FDG-PET/CT). Only diagnostic centres may refer directly to a ^18^F-FDG-PET/CT scan, and it remains controversial if CT or ^18^F-FDG-PET/CT is the most appropriate first-line modality in NSSC; for example, owing to diagnostic yield and false positive findings.^15,16^

The NSSC-CPP are organised slightly differently in each Danish region^17^ and data are sparse on basic demographics, symptoms, cancer prevalence, and imaging in the NSSC populations at the authors' hospital; this is currently debated between referring physicians and radiology departments.

The study objective was to contribute novel insights into patients on NSSC-CPP, including the distribution and significance of symptoms and the diagnostic yield of CT and ^18^F-FDG-PET/CT, with the aim of improving the overall work-up process.

## Method

This was a retrospective review of medical charts. We included all patients with novel NSSC referred from general practice, specialist practice, or local diagnostic centre for CT of chest and abdomen or ^18^F-FDG-PET/CT at our hospital from 1 January–31 December 2020. We excluded patients with known cancers, cancer of unknown primary, referrals to specific CPP, and suspected non-melanoma skin cancer.

The primary outcome was the proportion of patients with NSSC diagnosed with cancer. Any finding on a scan that was considered suspicious of cancer was classified as true or false positive or negative using the final diagnosis from the medical charts as reference standard.

We followed patients for a maximum of 1 year after the initial scan, but stopped in the event that they were diagnosed with cancer. Cancer diagnoses were verified from biopsy results in the Danish Pathology Register^18^ and were grouped according to International Classification of Diseases, Tenth Revision (ICD-10) chapters C00–D49. When no biopsy was performed despite suspicious scans, for example, owing to patient frailty, an experienced onco-radiologist re-analysed the scans to assess whether the findings were consistent with cancer.

Secondary endpoints included the most frequent initial symptoms and the number of subsequent examinations during the diagnostic work-up process.

The most frequent cancer symptoms the patients presented with were registered to their GP or diagnostic centre, and seven well-known warning signs of cancer; that is, long-term dysphagia, weight loss, long-term coughing or hoarseness, changed bowel habits, unexplained bleeding, changed moles or non-healing wounds, and changing lumps or swellings.^19^ The list was limited to 20 symptoms or signs plus ‘no symptoms’ and ‘doctor’s gut feeling’.

In addition, data were registered on subsequent examinations induced directly by the scan, that is, imaging, endoscopy, or biopsy performed within the follow-up period after initial referral, that is, for instance, control scans of known findings were not included.

Study data were collected and managed using REDCap (Research Electronic Data Capture), hosted by the Region of Southern Denmark.^20,21^

Statistic analysis was performed using Stata/BE (version 17), with continuous data reported by mean and standard deviation if normally distributed, and compared using Student’s *t*-test. Discrete or non-normal data were presented by median and range. Categorical data were represented as prevalence, and differences tested using χ^2^ or Fisher’s exact test. Statistical significance level was defined as 0.05.

## Results

**Figure 1.**
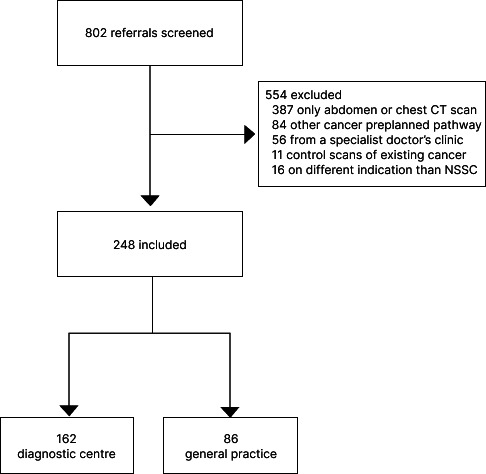
Flowchart showing enrolment of patients. CT = computed tomography. NSSC = non-specific symptoms or signs of cancer.

Of 802 referrals (178 from the diagnostic centre and 624 from GP or specialist practice), 248 matched the inclusion criteria (123 men and 125 women) (Figure 1). Table 1 presents baseline demographics and characteristics.

**Table 1.** Characteristics of patients (*n* = 248). Patients were divided into two groups depending on the referral site

	General practice (*n* = 86)	Diagnostic centre (*n* = 162)	*P* value
**Sex**			
Women, *n*	43 (50.0%)	82 (50.6%)	0.926^a^
Men, *n*	43 (50.0%)	80 (49.4%)	
**Age**			
Total, mean (± SD)	69.8 (±11.1)	67.3 (±14.4)	0.160^b^
**Modality**			
CT, *n*	85 (98.8%)	105 (64.8%)	**<0.000** ^a*^
^18^F-FDG-PET/CT, *n*	1 (1.2%)	57 (35.2%)	
**Cancer**			
Cancer, *n*	25 (29.1%)	27 (16.7%)	**0.009** ^a*^
Non-cancer, *n*	61 (70.9%)	135 (83.3%)	
Total	86	162	
**Number of symptoms**	2 (0–7)	2 (0–8)	**0.002** ^c*^
*Total*	86	162	

^a^χ^2^ test. ^b^ *t*-test. ^c^Fisher’s exact test. Bold and asterisked = statistically significant.

Findings indicative of cancer were found in *n* = 81/248 (33%) scans. Ultimately, *n* = 52/248 patients (21%) were diagnosed with cancer (Table 1).

**Figure 2.**
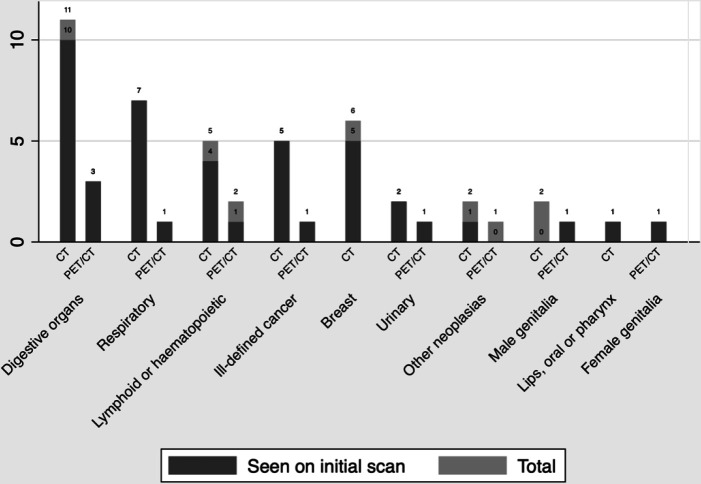
Types of cancers seen on initial scan grouped by the modality of the initial scan. A bar represents the number of cancer types detected in the group that was initially scanned using CT or ^18^F-FDG-PET/CT. The dark area shows how many of the cancers that were suspected on the initial scan (true positives). CT = computed tomography. PET/CT = positron emission tomography/computed tomography.

The most common cancer sites were the digestive organs (27% of 52), respiratory system (15%), and lymphoid or haematopoietic malignancies (14%) (Figure 2). Figure 2 illustrates the frequency of cancers and cancer suspicions in the initial scan; initial imaging detected 85% (*n* = 44/52) of cancers.

**Figure 3.**
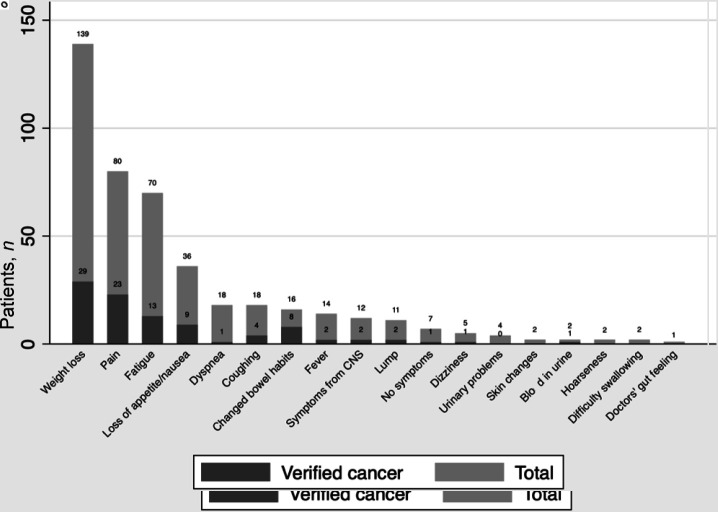
Overview of the symptoms reported by the included patients. The dark-coloured bars represent the proportion of patients with cancer who reported each symptom. Each patient may present with multiple symptoms. 'No symptoms' was used when the patient was referred owing to abnormal blood test results, suspicious findings on imaging, or any other finding that led the physician to refer the patient. CNS = central nervous system.

Patients presented to the referring physician with 0–8 symptoms with a median of 2 (Table 1). The most frequent symptom was weight loss (56% of the patients), and *n* = 78/248 (32%) had only one symptom. Some symptoms were more frequently associated with cancer: changed bowel habits (8/16), blood in the urine (*n* = 1/2), or pain (*n* = 23/80) (Figure 3). The second and third most common symptoms or findings in the present study were pain (32% overall, 9% of patients with cancer) and fatigue (28% overall, 5% of patients with cancer). When no symptoms were reported, the reasons for referral was usually abnormal results of blood samples or suspicious imaging findings.

After correlation of scans with final diagnoses, the diagnostic yield of initial CT and ^18^F-FDG-PET/CT was calculated. CT had a sensitivity of 85%, specificity of 87%, PPV of 65%, and NPV of 96%. For ^18^F-FDG-PET/CT, the numbers were sensitivity 82%, specificity 62%, PPV 33%, and NPV 94%. Full details of the diagnostic yield for each, before and after re-evaluation of scans, is presented in Table 2. Cancer prevalence was 22% (*n* = 41/190) in the CT group and 19% (*n* = 11/58) in the ^18^F-FDG-PET/CT group, respectively.

**Table 2.** Sensitivity, specificity, and predictive values of initial CT and ^18^F-FDG-PET/CT before and after re-evaluation of scans

Modality	Sensitivity (95% CI)	Specificity (95% CI)	PPV (95% CI)	NPV (95% CI)
**Original data**				
CT (*n* = 190)	85% (71% to 94%)	87% (81% to 92%)	65% (51% to 77%)	96% (91% to 98%)
^18^F-FDG-PET/CT (*n* = 58)	82% (48% to 98%)	62% (46% to 76%)	33% (17% to 54%)	94% (79% to 99%)
**After modifications**				
CT (*n* = 190)	87% (73% to 95%)	90% (84% to 94%)	72% (58% to 84%)	96% (91% to 98%)
^18^F-FDG-PET/CT (*n* = 58)	100% (66% to 100%)	63% (48% to 77%)	33% (17% to 54%)	100% (89% to 100%)

Modifications consisted of treating obvious cancer suspicions on CT as true positives and evaluating ^18^F-FDG-PET/CT on its ability to detect solid tumours (raw numbers are found in Supplementary data Table S1–S4).

CT = computed tomography. ^18^F-FDG-PET/CT = fluorine-18 fluorodeoxyglucose positron emission tomography/computed tomography. NPV = negative predictive value. PPV = postive predictive value.

Seven patients with findings suspicious of cancer on the initial CT scan had no biopsies. When CT scans were re-analysed, four of the patients had obvious cancer based on imaging alone and died during follow-up; these could arguably be classified as true positives.

Two non-solid haematological cancers (leukaemia) were not detected by ^18^F-FDG-PET/CT (Figure 2). These should be diagnosed by blood samples and not imaging. ^18^FFDG-PET/CT is suitable for the detection of solid tumours^22^ and hematologic cancers could be categorised as true negatives with respect to solid tumours.

The diagnostic yield after applying these modifications can be found in Table 2.

After the initial scan, patients often underwent additional examinations; patients without cancer went through 0–5 subsequent examinations (median of 1). Of those, 62 underwent one examination and 44 underwent more. CT scans and endoscopies were the most common supplementary examinations (Table 3); of all patients, 87 underwent endoscopies, in 40 cases with biopsy performed.

**Table 3.** Types of supplemental examinations each patient without cancer (*n* = 196) underwent grouped according to the initial imaging (CT or ^18^F-FDG-PET/CT)

Examination	CT group(*n* = 149)	^18^F-FDG-PET/CT group (*n* = 47)
CT	44 (29.5%)	14 (29.8%)
Endoscopy	12 (8.1%)	12 (25.5%)
MRI	13 (8.7%)	4 (8.5%)
^18^F-FDG-PET/CT	12 (8.1%)	2 (4.3%)
X-ray	7 (4.7%)	5 (10.6%)
Bone marrow biopsy	5 (3.4%)	2 (4.3%)
Ultrasound	5 (3.4%)	3 (6.4%)
Mammography	3 (2.0%)	2 (4.3%)
Other	19 (12.8%)	11 (23.4%)
*Total*	120	55

The category 'Other' included bronchoalveolar lavage, thyroid scintigraphy, and pleuro-/paracentesis. Data were registered from the Danish Pathology Register and the radiology information system of the hospital.

CT = computed tomography. ^18^F-FDG-PET/CT = fluorine-18 fluorodeoxyglucose positron emission tomography/computed tomography. MRI = magnetic resonance imaging.

## Discussion

### Summary

Twenty-one per cent of included patients were diagnosed with cancer, most frequently in the digestive organs, respiratory system, and lymphoid or hematopoietic system. The most common initial symptoms were weight loss, pain, or fatigue. CT and ^18^F-FDG-PET/CT had comparably high sensitivity and NPV, whereas CT had superior specificity and PPV.

### Strengths and limitations

This is a retrospective study with the inherent limitations of this design; for example, important data may be unavailable and data are prone to bias and confounders that are difficult to control. To minimise the risk of selection bias, all referrals to CT received as part of a NSSC-CPP at the authors' institution in 2020 were included regardless if they originated from general practice, specialist clinic, or diagnostic centre. Also, biopsy was used as the reference standard and cancer diagnoses were retrieved directly from the national pathology database to minimise the risk of misclassification and recall bias. This ensured a diagnosis was not missed even if a patient moved to a different region of Denmark.

On the negative side, the number of patients and scans in the dataset is relatively limited leading to some wide confidence intervals of all diagnostic properties (sensitivity, specificity, PPV, NPV) especially in the case of ^18^F-FDG-PET/CT. Thus, a misclassification would have a considerable impact on diagnostic properties.

Also, the study was observational with no interventions, meaning that the patients in the study were pre-selected for CT and ^18^F-FDG-PET/CT depending on the clinical setting and the referring physician's discretion. Therefore, the two groups were not directly comparable, although the cancer prevalence was similar in both groups.

Some patients were undoubtedly diagnosed with non-malignant diseases that were relevant as differential diagnoses in the context of NSSC, but owing to technical issues after a regional switch to a new electronic patient record, the authors did not have full access to historic electronic patient charts. Therefore, the study could not investigate this further or verify any post-scan clinical procedures or examinations except endoscopies.

Initial symptoms based on referral text were registered and there could be reporting bias if referring doctors disregarded some symptoms or findings in the referrals.

### Comparison with existing literature

The study found 21% had a biopsy proven malignancy. Another 2% (*n* = 4/248) had imaging findings in keeping with malignancy not confirmed by biopsy. Arguably, the prevalence is 23% and within the range in the literature. Møller *et al* found a prevalence of 20% in a cohort from general practice.^23^ Prevalence of cancer with NSSC in Denmark, Sweden, the UK, The Netherlands, Australia, and Spain were found to be 9%–35%.^13,14,23–30^ The COVID-19 pandemic stressed healthcare systems in 2020 and a general decrease in detected cancer incidence was observed.^31,32^ The prevalence of cancer in the present study is similar to studies before COVID-19^14,23,26,33^ and any influence of the COVID-19 pandemic could not be detected in the results.

In adherence to Danish General Data Protection Regluation (GDPR) legislation, the overall groupings of malignant findings had to be kept relatively broad; the most common cancer sites were the digestive organs (27%), respiratory system (15%), and lymphoid or haematopoietic malignancies (14%). These results were in keeping with the literature, for instance Chapman *et al* found the three most common cancers to be gastrointestinal cancers (upper and lower) (35%), lung (22%), and haematological (13%).^24^ Several other national and international studies found comparable results, albeit with variations in numbers.^25,26,29,34^

In agreement with other studies,^13,15,26,34^ more than half of patients (56% overall, 12% of those with a cancer diagnosis) were referred with weight loss. For instance, in a large English study, Chapman *et al* found weight loss in 66%, in 20% it was monosymptomatic.^24^ Unintended weight loss is associated with cancer, but not often explored in a standardised manner.^35,36^ Interestingly, it was recently discussed at the authors' institution whether monosymptomatic weight loss is enough to qualify a patient for the NSSC-CPP. Given the frequency of this symptom among patients with NSSC, weight loss may warrant further studies to investigate if it could predict cancer in itself. The second and third most common symptoms or findings in the present study were pain (32% overall, 9% of patients with cancer) and fatigue (28% overall, 5% of patients with cancer). Chapman *et al* found similar results; that is, pain and fatigue were the third and fifth most common (32% and 19%, respectively).^24^

Of note, 6% of the patients in the cohort presented with changed bowel habits, which would actually qualify them for the national colorectal CPP, and the reason why they entered the NSSC-CPP is unknown, but probably just signifies the complexity of this population.

The cohort consisted of patients with NSSC from both general practice and the diagnostic centre of the authors' institution; NSSC are common and can be a challenge to general practice.^34,36,37^ Organisation of CPP vary among institutions both nationally and internationally,^29,34,38,39^ and there is an ongoing effort to gather information to optimise the efforts.^37^

Imaging is routinely used in the diagnosis, staging, and follow-up of cancer, and although several studies addressed the diagnostic yield of advanced imaging in patients with NSSC, timing, first-line choice, and cost-effective use of imaging in patients with NSSC remains controversial.^13,23,29,33,37,40^ For instance, a current protocol is testing rapid CT in this context,^41^ and in Denmark there are disagreements over conventional CT or ^18^F-FDG-PET/CT as first-line modality.^15,16^

Initial scans detected 85% (*n* = 44/52) of cancers in the present study. Møller *et al* investigated the diagnostic properties of contrast-enhanced CT in NSSC-CPP referred from general practice. Cancer prevalence was 20%, and 92% had CT results classified as possible or probable cancer; a positive CT raised the probability of a cancer diagnosis to 62%, whereas a negative one decreased the probability to 2%.^23^ Similar results were reported by Ormstrup *et al*.^33^

^18^F-FDG-PET/CT has been suggested instead of CT as initial imaging in NSSC for more timely diagnoses and cost-effectiveness.^15,16^ The results found that CT and ^18^F-FDG-PET/CT had comparable and reasonable sensitivity and high NPV. CT had better specificity and higher PPV than ^18^F-FDG-PET/CT; that is, PPV of 65% for CT versus 33% for ^18^F-FDG-PET/CT. Thus, ^18^F-FDG-PET/CT was as sensitive as CT and as effective in ruling out cancer suspicion but induces further examinations, as well as still being less available and more expensive. These results are consistent with other studies;^15,16,42^ although Lebach *et al* found higher PPV for ^18^F-FDG-PET/CT than CT, it was not statistically significant. They also classified ^18^F-FDG-PET-positive lesions without clear anatomic CT substrate as negative for cancer, and ^18^F-FDG-PET-negative but malignant-looking tumour on the CT part of ^18^F-FDG-PET/CT as positive for cancer on FDG-PET. By doing so, they may have inadvertently introduced a bias by removing false positive cases from ^18^F-FDG-PET/CT and introducing more false positives on CT.

Most malignant diagnoses in the present study were established during the initial work-up process, but in two cases, the diagnoses were not established until 6 months and 1 year, respectively, after referral. One patient presented with fatigue, weight loss, and gastrointestinal symptoms and was diagnosed with metastatic breast cancer 150 days later. The other presented with fatigue and dizziness and findings in keeping with arteritis on ^18^FFDG-PET/CT. This patient was diagnosed with chronic lymphocytic leukaemia 300 days later. It is unclear if their symptoms were related to their final malignant diagnosis.

Suspicion of prostate cancer was raised twice on ^18^F-FDG-PET/CT but not reported as suspicious on CT (Figure 2). Hypertrophy of the prostate was reported in both patients with prostate cancer, but CT is not considered diagnostic in the routine work-up for prostate cancer.^43^

Some patients were initially CT scanned before entering the NSSC-CPP and were subsequently referred for ^18^F-FDG-PET/CT. Generally, patients without cancer went through multiple examinations, such as endoscopy, illustrating the diagnostic challenges in this patient group. More than half of the patients without cancer underwent at least one additional examination after the initial scan before the work-up process was concluded. Other studies showed a similar trend.^16,44^ ^18^F-FDG-PET/CT may identify incidental gastrointestinal findings potentially representing malignancy,^45^ thus, patients without cancer with initial ^18^F-FDG-PET/CT underwent relatively more endoscopies compared with those with initial CT.

### Implications for research and practice

The findings in patients on NSSC-CPP were in accordance with the literature. A cancer prevalence of 21% was found, most frequently in the digestive organs. The most frequent symptom was weight loss as reported by more than half of the patients and, even as the only symptom, it is a potential marker for cancer. CT and ^18^F-FDG-PET/CT were sensitive with high NPV, whereas PPV was superior in CT. Patients without a cancer diagnosis underwent subsequent examinations following initial imaging with CT or ^18^F-FDG-PET/CT.

The data have underlined the heterogeneous presentation of patients with NSSC, with a multitude of potential symptoms and findings. The data also support the current imaging strategy in NSSC-CPP. CT and ^18^F-FDG-PET/CT both have a place, but based on the study data, it was not possible to establish why patients were referred for CT or ^18^F-FDG-PET/CT. Future, prospective studies are needed to better stratify patients according to presentation to further optimise the NSSC-CPP work-up strategy.

## Data

According to Danish legislation on personal health data and GDPR, it is not permitted to make the complete dataset available.
